# Metagenome and Metatranscriptome Profiling of Moderate and Severe COPD Sputum in Taiwanese Han Males

**DOI:** 10.1371/journal.pone.0159066

**Published:** 2016-07-18

**Authors:** Shih-Wei Lee, Chin-Sheng Kuan, Lawrence Shih-Hsin Wu, Julia Tzu-Ya Weng

**Affiliations:** 1 Taoyuan General Hospital, Ministry of Health and Welfare, Taoyuan, Taiwan; 2 Department of Life Sciences, National Central University, Taoyuan, Taiwan; 3 Institute of Medical Sciences, Tzu Chi University, Hualien, Taiwan; 4 Department of Computer Science and Engineering, Yuan Ze University, Taoyuan, Taiwan; 5 Innovation Center for Big Data and Digital Convergence, Yuan Ze University, Taoyuan, Taiwan; Queens University Belfast, IRELAND

## Abstract

Chronic obstructive pulmonary disease (COPD) is an inflammatory lung disorder characterized by the progressive obstruction of airflow and is currently the fourth leading cause of death in the world. The pathogenesis of COPD is thought to involve bacterial infections and inflammations. Owing to advancement in sequencing technology, evidence is emerging that supports an association between the lung microbiome and COPD. However, few studies have looked into the expression profile of the bacterial communities in the COPD lungs. In this study, we analyzed the sputum microbiome of four moderate and four severe COPD male patients both at the DNA and RNA level, using next generation sequencing technology. We found that bacterial composition determined by 16S rRNA gene sequencing may not directly translate to the set of actively expressing bacteria as defined by transcriptome sequencing. The two sequencing data agreed on *Prevotella*, *Rothia*, *Neisseria*, *Porphyromonas*, *Veillonella*, *Fusobacterium* and *Streptococcus* being among the most differentially abundant genera between the moderate and severe COPD samples, supporting their association with COPD severity. However, the two sequencing analyses disagreed on the relative abundance of these bacteria in the two COPD groups, implicating the importance of studying the actively expressing bacteria for enriching our understanding of COPD. Though we have described the metatranscriptome profiles of the lung microbiome in moderate and severe COPD, further investigations are required to determine the functional basis underlying the relationship between the microbial species in the lungs and pathogenesis of COPD.

## Introduction

Chronic obstructive pulmonary disease (COPD) is an inflammatory lung disorder characterized by the progressive obstruction of the airway, which leads to symptoms such as shortness of breath, cough, wheezing, dyspnea, as well as increased sputum production [1, 2]. In 2004, COPD was reported to be the fourth leading cause of death, accounting for 5.1% of global mortality [3]. It is projected that by 2020, COPD will become the third and fifth leading cause of global deaths and disability, respectively [4].

The progressive limitation of airflow in COPD is generally irreversible, often leading to small airway fibrosis and emphysema, destroying alveolar space in addition to causing mucus hyper-secretion [1]. Exacerbations of COPD can result in high mortality and morbidity, rapid decline in lung function, and increased health care expenses [5]. Smoking [6], air pollution [7, 8], viral infection [9], as well as bacterial infections such as tuberculosis [10–12], are major contributors of COPD risk. Though cigarette smoking is associated with COPD, not all smokers develop the disease [1, 2]. Furthermore, exacerbations can develop, but not all patients are susceptible to the symptoms. COPD is, therefore, a heterogeneous disease that may be affected by multiple factors which are not yet fully understood.

The pathogenesis of COPD is thought to involve inflammatory mediators and infections [13]. It is estimated that 50 to 60% of exacerbations are due to bacterial or viral infections [14]. In particular, systemic [15] and airway inflammation [16] are often associated with exacerbations. Traditional microbial culturing techniques have already found evidence of bacterial colonization in the airways of COPD patients between exacerbations [17]. These bacteria persist in the respiratory tract, making up the lung microbiome. The extent to which exacerbations are influenced by the presence of microbes is not yet clearly defined, but it is postulated that any pathogen exposure may increase the patient’s susceptibility to chronic inflammation, worsening respiratory symptoms and accelerating disease progression [18].

Despite the hypothesis that microbial infection may play a role in COPD, the nature of this relationship and the significance of bacteria-induced inflammations remain controversial. What is known is that bacterial infection in the lower respiratory tract are common in COPD patients, and this intense bronchial inflammation may continue even after the patients have stopped smoking [19]. Furthermore, this inflammation is not only evident in patients with severe COPD, but also present in moderate COPD patients who show relatively mild symptoms [20]. Both cases can lead to accelerated and sustained loss of lung function [16, 21].

Corticosteroids are standard treatment for COPD exacerbations. Combined with antibiotics or anti-inflammatory agents, the therapy has demonstrated to be effective in most cases [22]. However, the continuous use of antibiotics has no effect on reducing the frequency of exacerbations [23, 24]. Moreover, one of the side-effects of corticosteroid is weakening of the host defense, leading to increased risk of infection and pneumonia [25]. In animal experiments, promising results were seen in the use of probiotics to reduce lung inflammation, though human studies have yet to reach a conclusive finding [26]. Regardless, it is evident that bacteria play a role in the progression of COPD, and the microbial composition in the lung, or microbiome [27], may provide insights into the effect of bacterial infection on lung function and offer new perspectives to the current understanding of COPD pathogenesis.

Numerous bacterial species occupy specific niches in the human body and share a symbiotic relationship with their host [27]. Contrary to traditional beliefs, the lower respiratory tract is not sterile [28]. Moreover, though not all available literature agrees on the role of bacteria in the pathogenesis of COPD exacerbations, possibly due to the heterogeneous nature of COPD conditions and the questionable effectiveness of antibiotics, indirect evidence of chronic bronchitis, respiratory infections and sputum bacterial counts has demonstrated the association between bacteria and decline in lung function [29].

The advent of next generation sequencing (NGS) technology brings the promise of more advanced sensitivity and efficiency in detecting a broad range of microbial species harboring the lung environment. Compared to traditional sequencing method, NGS provides a cost-effective, high-throughput method to directly scan the genome or transcriptome of organisms in order to obtain a large number of short nucleotide sequence reads in a short time [30]. With limited knowledge about the underlying nature of lung microbiome in the progression of COPD, NGS facilitates efficient and sensitive capture of the microbial inhabitants in the lung.

Already, it has become apparent that the spectrum of bacteria present in the lungs of COPD patients differs from those with normal lungs [31, 32]. Several recent studies have attempted to describe the lung microbiome by using NGS to sequence the highly conserved 16S ribosomal RNA (16S rRNA) genes that are present in bacteria but absent in their human hosts [31–33]. For instance, increased bacterial diversity was found in COPD patients compared to controls [33]. In contrast to moderate COPD patients, more Firmicutes and fewer Actinobacteria and Proteobacteria were found in severe COPD patients [32]. Differences in the COPD lung microbiome may lead to altered immune tolerance and inflammatory state of the airway, influencing the progression of COPD.

To our knowledge, very few studies have investigated the metatranscriptome of the lung microbiome among patients with differing severity of COPD. The metatranscriptome represents the gene expression profiles of the microbial species in the lung. In addition to understanding the composition of the microbiome in the COPD lungs, knowledge about the types of bacteria that are actively expressing their genomes offers more direct insights to the roles that these bacteria play in the differences between severe and moderate COPD.

In this study, we applied the NGS technology to sequence the bacterial 16S rRNA genes and transcriptome in the sputum samples acquired from severe and moderate COPD male patients. Results from the 16S rRNA and metatranscriptome sequencing were compared and used to reflect the composition of the lung flora in patients with moderate and severe COPD. Our study uncovered bacteria that may be active within the lungs in these two COPD stages and help establish a better understanding of the lung microbiota associated with COPD.

## Methods and Materials

### Clinical sample collection

All procedures performed in this study have been approved by the Institutional Review Board of Taoyuan General Hospital, Ministry of Health and Welfare, Taiwan. Written informed consent was obtained from each participant. Diagnosis and classification of COPD were established according to GOLD (Global Initiative for Chronic Obstructive Lung Disease) [34] recommendations. Sputum samples were acquired from eight male participants: four with moderate COPD (GOLD 2) and four with severe COPD (GOLD 3) in a stable condition (at least 3 months without exacerbation or use of antibiotics for any other reason). The patients were being treated with long-acting beta-agonist (LABA), long-acting muscarinic antagonist (LAMA), inhaled corticosteroid (ICS) or a combination of at least two of these prescribed medications. Clinical data, such as weight, height, body mass index (BMI), history of smoking, presence of other diseases or disorders, forced expiratory volume in 1 second (FEV1), forced vital capacity (FVC), etc. were collected.

### DNA and RNA extraction

Sputum samples were collected over the period of two weeks during spring of 2015. Approximately 2 ml from each patient, were collected into 50 ml BD Falcon^™^ tubes, mixed with 6 ml of Trizol^®^ LS (ThermoFisher Scientific, MA, USA), stored in Trizol^®^ LS at -70°C, until all samples were ready for subsequent DNA and RNA isolation following the manufacturer’s protocol. Quality and quantity of the extracted DNA and RNA were assessed on Implen’s NanoPhotometer^®^ Pearl (München, Germany), and subsequently visualized by performing 2% agarose gel electrophoresis. RNA integrity was examined on the BioRad Experion system (CA, USA). Sample processing and subsequent sequencing reactions were performed in the same batch, using the same respective kits, by the same technician, and at the same laboratory setting at Health GeneTech Corporation, Taoyuan, Taiwan.

### Library preparation and sequencing

DNA samples were prepared with Truseq Nano DNA LT (Illumina, CA, USA). Sequence of the polymerase chain reaction (PCR) primers for amplifying the V4 domain of bacterial 16S rRNA were designed according to [35]: F515 (5′-GTGCCAGCMGCCGCGGTAA-3′) and R806 (5′-GGACTACHVGGGTWTCTAAT-3′). Amplification was conducted in a 50 μl reaction volume containing 25 μl of 2X Phusion Flash Master Mix (ThermoFisher Scientific, MA, USA), 0.5 μM each of the forward and reverse primer, and 50 ng of the DNA template. A negative control of the same reaction volume containing double-distilled H_2_O was included. The reaction began with initial 30 seconds at 98°C, followed by 30 cycles of 10 seconds at 98°C, 30 seconds at 54°C, 72°C for 30 seconds, and a final extension of 5 minutes at 72°C. The amplicons were first examined by 2% agarose gel electrophoresis and ethidium bromide staining, and subsequently purified using the Agencourt AMPure XP PCR Purification Kit (Beckman Coulter, Inc. CA, USA). Quantification of the purified amplicons were performed using a Qubit dsDNA HS Assay Kit (ThermoFisher Scientific, MA, USA) on a Qubit 2.0 Fluorometer (ThermoFisher Scientific, MA, USA). The V4 libraries were prepared by attaching Illumina adapters to the amplicons using TruSeq DNA Sample Preparation v2 Kit (Illumina, CA, USA). The libraries were purified and applied for cluster generation prior to being sequenced on the Miseq system (Illumina, CA, USA). Total RNA, 400 ng per sample, was subjected to Epicenter’s ScriptSeq^™^ Complete Kit (WI, USA) for rRNA depletion and library preparation involving cDNA synthesis, 5’- and 3’-tagging, as well as index PCR. Quality and quantity of the prepared libraries were assessed by qPCR and on a Qubit 2.0 Fluorometer (ThermoFisher Scientific, MA, USA) prior to being sequenced on the NextSeq 500 platform (Illumina, CA, USA). Negative controls were not performed in the extraction process, but a no-template negative control was included in the library preparation and subjected to 16S rRNA gene sequencing. Also, because no RNA was detected in this sample, it was not included in subsequent RNA sequencing reactions.

### Quality filtering of sequencing Data

**Fig 1:**
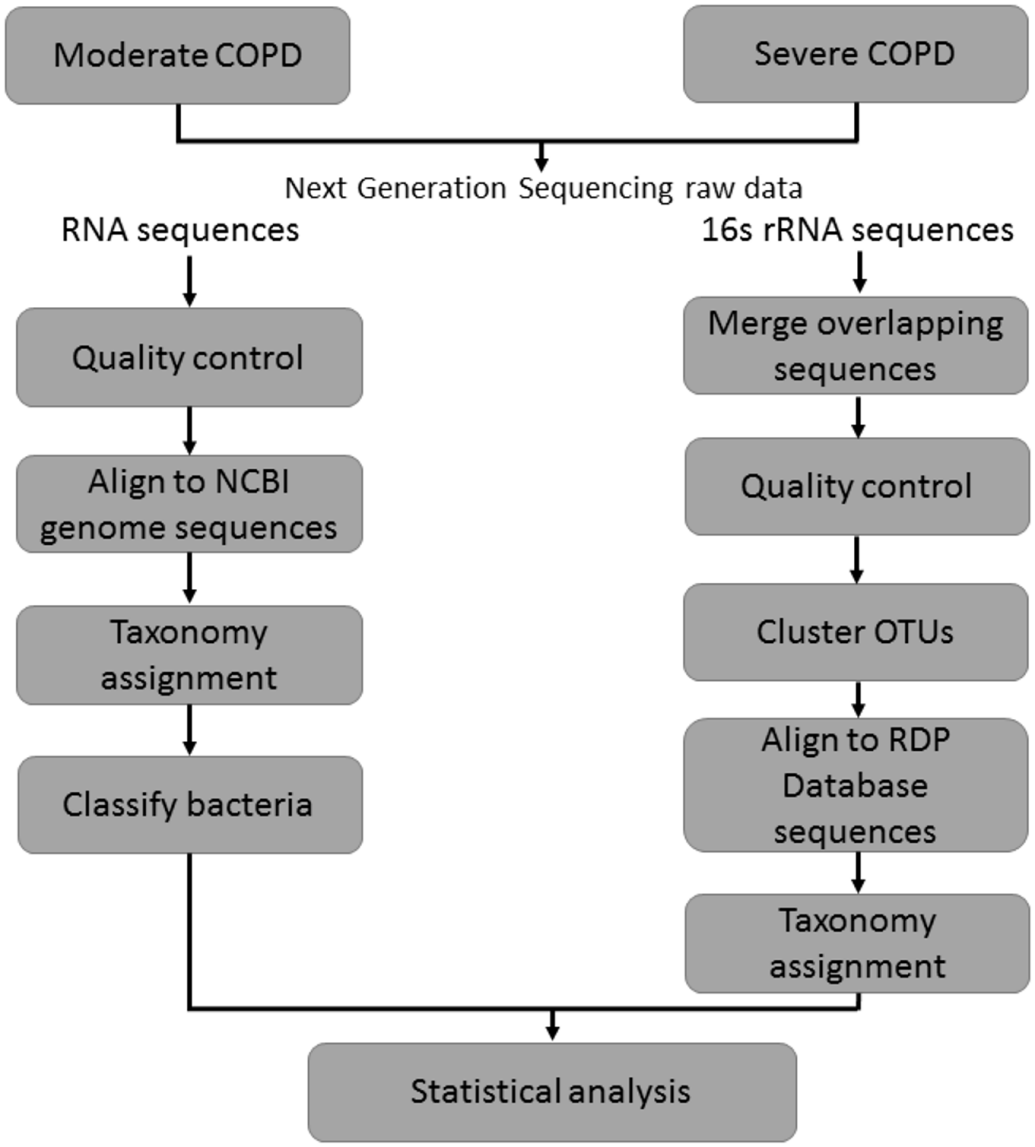
Analytical flow of our study.

Raw sequencing data are available on Harvard Dataverse via https://dataverse.harvard.edu/dataverse/twCOPD and in the NCBI Sequence Read Archive (SRA) via project accession number PRJNA322414 for 16S rRNA gene and transcriptome sequence datasets, respectively Raw sequencing data are available on Harvard Dataverse via https://dataverse.harvard.edu/dataverse/twCOPD and in the NCBI Sequence Read Archive (SRA) via project accession number PRJNA322414 for 16S rRNA gene and transcriptome sequence datasets, respectively. Preprocessing of the raw reads is illustrated in Fig 1. Raw Paired-end sequences and single end sequences in FASTQ format were generated from 16S rRNA gene sequencing and RNA sequencing, respectively. Overlapping paired-end 16S rRNA gene sequences were merged using UPARSE [36]. Then, the FASTX-Toolkit (version.0.013; http://hannonlab.cshl.edu/fastx_toolkit) was utilized for quality assessment of both raw 16S rRNA and RNA sequence datasets. First, raw sequences were processed to identify samples based on their corresponding barcodes from the 5’ end of the sequence with two allowed mismatches. The barcodes were trimmed, and low quality reads, filtered. High quality reads were obtained based on the following procedures: 1) reads that exceeded a minimum Phred quality score of 20 were retained; 2) sequences shorter than 70 bp were excluded; 3) reads containing ambiguous characters were discarded. A Phred quality score is a measure of the quality of base- calling and a score of 20 corresponds an error probability of 0.01, according to the formula: Q = −10log_10_*P*.

### Taxonomy assignment

The workflow of our taxonomy analysis is shown in Fig 1. UPARSE [36] was used to cluster high quality bacterial 16S rRNA gene sequences into operational taxonomic units (OTUs) by aligning high quality reads against a collection of 16S rRNA gene sequences extracted from the Ribosomal Database Project (RDP) database [37] which provides aligned and annotated 16S rRNA gene sequences from the National Center for Biotechnology Information (NCBI) database. A standard 90% sequence similarity against the database sequences was applied for the alignment. OTU assignments with fewer than 10 sequences were discarded. For taxonomy assignment based on high quality RNA sequences, Bowtie2 [38] was used to align these sequences against reference genome data built from the NCBI nucleotide (nt) database data, which was available as a file with the filename “nt.##.tar.gz” on the NCBI BLAST FTP site under the “/blast/db/” subdirectory (last downloaded on May 24, 2015). This dataset included sequence entries from GenBank, Ensembl, DNA Data Bank of Japan, etc. Sequences successfully mapped to the reference genome data with ≥95% similarity were classified into kingdom, phylum, class, order, family, genus, and species according to the corresponding taxonomic information obtained from NCBI. For the 16S rRNA dataset, reads in each sample were corrected against those in the negative control sample to minimize background noises or potential reagent contaminations. This adjustment was based on the relative abundance of the bacterial communities in the negative control sample. For example, if the reads mapped to the genus *Shewanella* accounted for 20% and 2% of all the detected bacteria in a patient’s sample and the negative control, respectively, then the relative abundance of *Shewanella* in the patient sample after correction would be 20 x (1–0.02) = 19.6%.

In addition, to examine bacterial communities common to all samples within the same group (i.e. moderate or severe COPD) and reduce the influence of individual differences, separate analyses were performed on unique 16S rRNA and RNA sequence reads. We defined unique reads as those that were mapped to one specific NCBI gi number with ≥90% and ≥95% sequence similarity for 16S rRNA and RNA sequence reads, respectively. Secondly, only those high-quality reads belonging to a specific genus and were detectable in every individual sample of the same COPD group were selected. For each uniquely mapped genus, coefficient of variation (CV) within each group was computed by following the formula: CV = *σ*/*μ*. The results were distributed across four quantiles. Genera with CV in the first quantile were considered to exhibit relatively minimal variations within each COPD group. For these genera, the differences in sequence reads between the moderate and severe patients were calculated and ranked in a descending order based on the extent of the differences.

## Results

To investigate the bacterial communities in the sputum of moderate and severe COPD, eight samples (4 moderate and 4 severe COPD males) were subjected to 16S rRNA and transcriptome sequencing to profile the metagenomes and metatranscriptomes associated with the different COPD severities. The two groups did not differ significantly in age or past smoking statuses (Table 1; Wilcoxon rank sum test, p>0.05). S1 Table provides more detailed clinical data of these patients. While no statistically significant differences in other clinical characteristics were found between the two groups (Wilcoxon rank sum test, p>0.05), patients from both groups were being treated with long-acting beta-agonist (LABA), long-acting muscarinic antagonist (LAMA), inhaled corticosteroid (ICS) or a combination of at least two of these prescribed medications, at the time of sample collection.

**Table 1:** Clinical data of the male moderate and severe COPD participants.

Participants	Age	History of smoking	FEV1	FVC	FEV1/FVC
Moderate 1	70	N	2.24	2.96	76
Moderate 2	71	Y	1.04	1.75	60
Moderate 3	72	Y	1.23	2.12	58
Moderate 4	57	Y	1.87	3.33	56
Severe 1	69	Y	1.8	3.72	48
Severe 2	91	N	0.66	1.61	41
Severe 3	78	Y	0.93	2.71	34
Severe 4	71	Y	1.27	3.92	32

Results of 16S rRNA gene sequencing are presented in Table 2. A total of 419,990 raw reads were obtained from all patient samples, and 91,697, from the negative control. After quality control filtering, 200,751 and 91,496 reads remained in the patient samples and negative control, respectively. Of the remaining high quality sequences, 107,707 patient sample reads and 63,632 negative control reads successfully mapped to the RDP reference data [37] with a minimum 90% sequence similarity. According to OTU clustering by UPARSE [36], 61 OTUs were identified in the negative control sample, while an average of 163 and 157 OTUs were found in the moderate and severe COPD group, respectively. No significant differences in the number of OTUs per sample were observed between the moderate and severe groups (Wilcoxon rank sum test, p>0.05). Shannon index was calculated for each sample. Though slightly higher diversity was observed in the average Shannon index of the severe group, it was not significantly different from the moderate group. Shannon index for the negative control sample was calculated to be 0.77. This negative control sample was specific to the library preparation process for the 16S rRNA gene sequencing. Therefore, only potential contaminants from this procedure were accounted for in the 16S rRNA gene analysis.

**Table 2:** 16S rRNA gene sequencing results for the bacterial metagenome in the sputum of moderate and severe COPD subjects.

Participants	Raw reads	High-quality reads	Mapped reads	Corrected reads	OTUs	Shannon Index	Corrected Shannon Index
Moderate 1	49,760	25,597	14,312	3,718	105	1.78	1.66
Moderate 2	83,694	47,387	25,733	17,301	381	2.69	2.65
Moderate 3	26,473	12,615	6,006	1,798	93	1.97	2.01
Moderate 4	34,861	19,059	10,926	2,642	73	1.47	1.45
Moderate COPD average	48,697±25,241	26,165±15,108	14,244±8,384	6365±7333	163±146	1.98±0.52	1.94±0.53
Severe 1	60,355	24,008	10,736	7,605	178	2.40	2.31
Severe 2	25,345	22,870	12,714	9,954	251	2.31	2.30
Severe 3	68,394	11,715	5,392	3,385	82	1.95	1.93
Severe 4	71,108	37,500	21,888	17,427	117	1.65	1.60
Severe COPD average	56,301±21,136	24,023±10,559	12,683±6,872	9593±5888	157±74	2.08±0.34	2.04±0.34

Average values are listed as average ± standard deviation.

**Fig 2:**
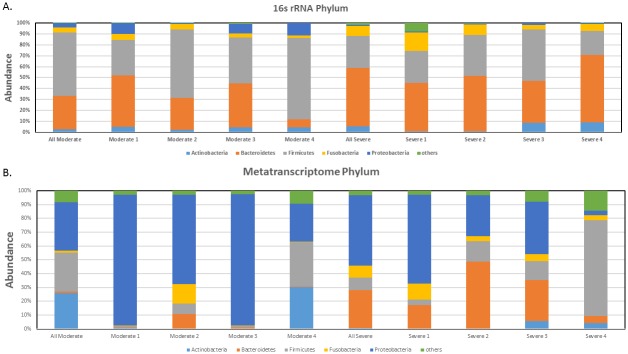
Relative sequence abundance among different phyla. A) Relative sequence abundance of bacterial phyla as identified by 16S rRNA gene sequencing. B) Relative sequence abundance of bacterial phyla as identified by metatranscriptome sequencing.

Taxonomic classifications, at the phylum and genus level, for each sample are provided in S2 Table. The most common phylum in the moderate group was Firmicutes, followed by Bacteroidetes, Fusobacteria, and Proteobacteria (Fig 2A). While the same four phyla were also present in the severe COPD group, the order in relative abundance was different, where Bacteroidetes was the most common, followed by Firmicutes, Fusobacteria, and Actinobacteria. Proteobacteria appeared to be reduced in severe COPD. Though Proteobacteria constituted a large proportion of the bacterial community in the negative control sample, the dominant species were *Halomonas* and *Shewanella*, which were extremely rare in the COPD samples (S2 Table).

**Fig 3:**
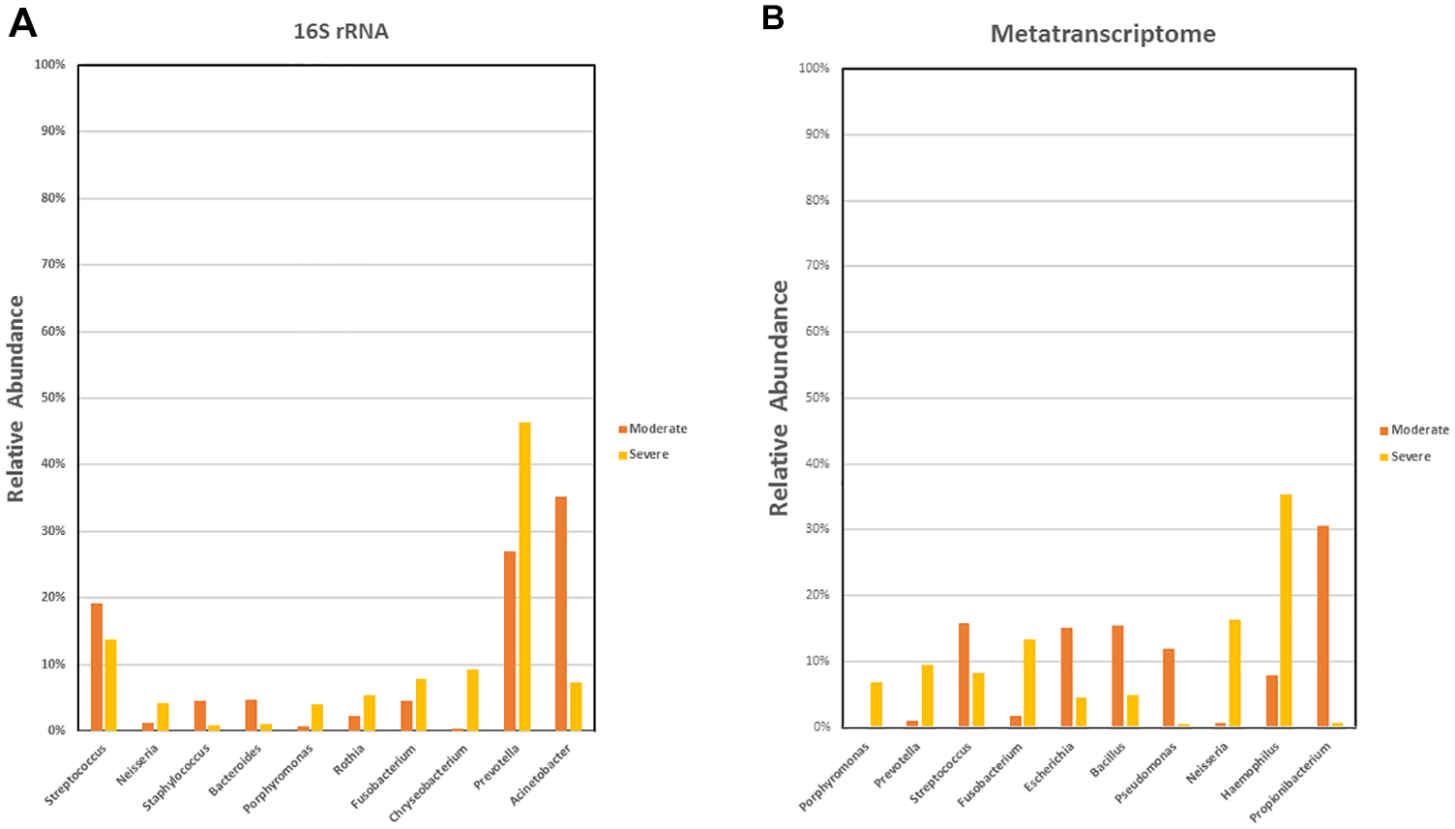
Top 10 genera showing the most difference in relative sequence abundance at the genus level in the moderate and severe patients. A) Top 10 most different genera in relative 16S rRNA sequence abundance. B) Top 10 most different genera in relative transcript sequence abundance.

With respect to the distribution of sequences across different taxonomic classifications, no significant differences were found between the moderate and severe COPD group from the phylum to the genus level (Wilcoxon rank sum test, p>0.05). There were, nevertheless, observable differences in sequence distribution. We calculated the percentage difference in sequence distribution between the moderate and severe group at the genus level. The resulting values were used to rank the genera in a descending order to identify the top 10 genera showing the greatest difference between the two COPD severities (Fig 3A; S3 Table).

Major difference was observed in the relative abundance of bacteria belonging to the genus *Prevotella and Acinetobacter*, in that the former is more, and the latter, less abundant, in the severe COPD samples compared to their moderate counterparts. Also, bacteria under the genera *Fusobacterium*, *Rothia*, *Porphyromonas*, *Neisseria*, and *Chryseobacterium* were more prevalent in the severe group. On the other hand, in the moderate COPD sputum samples, higher relative abundance of *Streptococcus*, *Staphylococcus*, and *Bacteroidetes* were apparent. Many of these bacteria that showed greater distinctions between the moderate and severe samples belonged to Bacterioidetes, Proteobacteria, and Firmicutes, three of the more dominant phyla in both groups.

**Fig 4:**
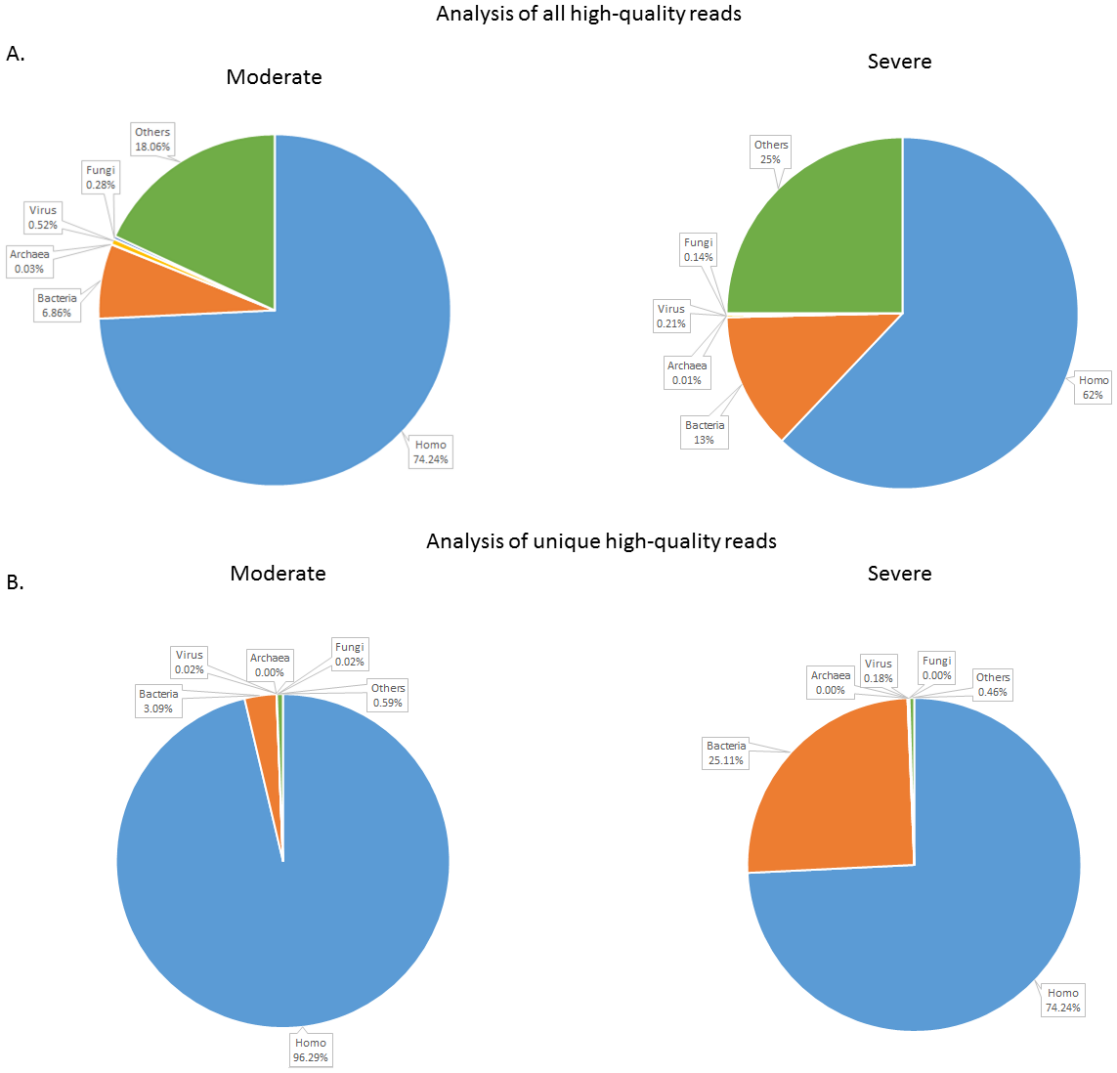
Sequence distribution among different kingdoms at the transcriptome level. A) Percent sequence distribution among different kingdoms as identified by analysis of all high-quality reads. B) Percent sequence distribution among different kingdoms as identified by analysis of high-quality unique reads.

Sequencing of the sputum RNA yielded a total of 383,737,281 raw reads, from which 287,853,580 high quality reads were extracted (Table 3). After aligning the reads against the NCBI reference genome data (built from the NCBI nucleotide (nt) database data which were last downloaded on May 24, 2015) with a similarity threshold of ≥95%, 248,899,146 sequences remained. While most of the reads were mapped to the human genome (74.24% and 62% for the moderate and severe COPD group, respectively), on average 9.93% of the reads appeared to belong to bacteria, 0.02% to archaebacteria, 0.37% to viruses, 0.37% to fungi, and the remaining sequences unrelated to humans or other kingdoms were assigned to the category of “others” (Fig 4). Though the relative abundance of bacteria seemed to be greater in the severe group, the moderate and severe COPD samples appeared to be statistically similar in sequence distribution across different kingdoms, as well as among various bacterial phyla, classes, families, and down to the species level (Wilcoxon rank sum test, p>0.05). Detailed sequence statistics are provided in S4 Table. Shannon index for each sample was also calculated. Similar to the 16S rRNA gene sequencing results, diversity among the severe group was greater than their moderate counterparts, though, again, the difference was not statistically significant.

**Table 3:** Metatranscriptome sequencing results for the bacterial metagenome in the sputum of moderate and severe COPD subjects.

Participants	Raw reads	High-quality reads	Mapped reads	Human (read counts)	Bacteria (read counts)	Archaea (read counts)	Virus (read counts)	Fungi (read counts)	Others (read counts)	Shannon Index
Moderate 1	45,555,205	41,642,413	38,894,741	34,490,886	211,591	30,495	1,794	9,333	4,150,642	0.48
Moderate 2	40,292,441	32,981,685	30,470,762	20,904,452	807,492	7,049	3,737	43,800	8,704,232	2.27
Moderate 3	45,334,008	41,793,501	40,182,052	38,201,059	32,935	4,368	1,458	909	1,941,323	0.48
Moderate 4	73,477,585	21,741,202	16,552,345	7,385,255	4,001,802	3,217	342,982	156,386	4,662,703	2.18
Moderate COPD average	51,164,810±15,072,428	34,539,700±9,474,441	31,524,975±10,871,207	25,245,413±14,037,530	1,263,455±1,855,357	11,282±12,909	87,493±170,329	52,607±71,631	4,864,725±2,818,914	1.35±1.01
Severe 1	49,577,314	45,066,829	35,038,934	16,521,576	807,492	543	5,960	75,889	12,565,610	2.26
Severe 2	44,179,073	28,706,855	16,435,822	7,617,886	5,313,659	886	18,288	22,396	3,462,707	2.26
Severe 3	42,193,436	37,742,565	35,813,197	21,650,789	301,170	6,408	258,223	69,756	13,526,851	2.59
Severe 4	43,128,219	38,178,530	35,511,293	33,489,840	124,862	11,275	2,364	1,600	1,881,352	1.34
Severe COPD average	44,769,511±3,306,236	37,423,695±6,709,999	30,699,812±9,514,663	19,820,023±10,801,042	1,636,796±2,468,259	4,778±5,097	71,209±124,863	42,410±36,215	7,859,130±6,037,008	2.11±0.54

Average values are listed as average ± standard deviation.

At the transcript level, bacterial communities in the moderate COPD sputum samples were dominated by Proteobacteria, Firmicutes, and Actinobacteria in descending order of mapped read counts (Fig 2B; S4 Table). In the severe group, the most common phylum appeared to be Proteobacteria, followed by Bacteroidetes, Firmicutes, and Fusobacteria. Note that one sample from each of the moderate and severe group were almost completely dominated by Proteobacteria, and one severe sample was highly enriched in Firmicutes.

Despite the lack of significant differences between moderate and severe COPD in taxonomic classifications, we still calculated the percentage difference in sequence distribution between the two groups at the genus level. Fig 3B illustrates the top 10 genera showing the most difference between the two groups. *Haemophilus*, *Neisseria*, *Fusobacterium*, *Prevotella*, and *Porphyromonas* seemed to be more abundant in the severe group, while the moderate samples were more enriched with *Propionibacterium*, *Bacillus*, *Pseudomonas*, *Escherichia*, and *Porphyromonas*. Most of these bacteria belonged to the phyla Proteobacteria, Bacteroidetes and Firmicutes. A detailed list of the ranking is provided in S5 Table.

At the phylum level, sputum 16S rRNA gene and RNA sequencing appeared to have identified different microbiome profiles for the moderate and severe COPD group. For instance, Firmicutes, and Bacteroidetes were more dominant in the moderate group as indicated by 16S rRNA gene sequence analysis, while RNA sequencing identified Proteobacteria and Actinobacteria to be the more highly represented phyla in the moderate samples (Fig 2; S4 Table). Moreover, metatranscriptomic analysis showed that Actinobacteria, determined by 16S rRNA gene sequencing as being present in relatively small amount, were more abundant than Bacteroidetes. On the other hand, both analytical approaches seemed to agree on Bacterioidetes and Firmicutes being among the more dominant phyla among the severe samples, but compared to the 16S rRNA gene sequence analysis, RNA sequencing detected increased and decreased relative abundance of Proteobacteria and Actinobacteria, respectively, in the severe group.

Discrepancies between 16S rRNA gene and RNA sequencing were even greater at the genus level (Fig 3; S5 Table). With respect to the top 10 genera showing the greatest differences in percentage sequence distribution among genera between the moderate and severe group, the two analytical methods disagreed on all except that relative abundance of bacteria belonging to *Prevotella*, *Fusobacterium*, *Porphyromonas*, and *Neisseria* were greater in the severe samples, while *Streptococcus* appeared to be more abundant in the moderate samples. Some of these differences between the severe and moderate group were, however, more evident in the metatranscriptomic analysis. Also, although *Staphylococcus*, *Bacteroides*, and *Acinetobacter* were listed as one of the top 10 most different genera between the moderate and severe group by 16S rRNA gene sequencing in terms of relative abundance, the same observations could not be repeated through the RNA sequencing analysis.

Due to the small sample size, individual variations might have prevented us from discovering statistically significant differences between the two COPD severities. Thus, to minimize individual influences, we decided to perform additional analyses targeting those reads that were mapped to only one specific species and common to all individuals of the same group at the genus level. We define these uniquely mapped high-quality reads as “unique reads,” that is, unique to a specific group of COPD severity. The unique reads consisted of 78.26% and 79.34% of the total high quality reads in the 16S rRNA gene sequence (S2 Table) and metatranscriptome dataset (S3 Table), respectively. No significant differences in kingdom distribution could be seen between the moderate and severe COPD group (Fig 4B). Note, however, unique sequence distribution among different kingdoms differed from the previous analysis that was based on all high-quality reads (Fig 4A), and compared to the moderate group, greater relative abundance of bacteria could be observed in the severe samples. However, this observable difference between the two COPD groups was statistically significant (Wilcoxon rank sum test, p>0.05).

**Fig 5:**
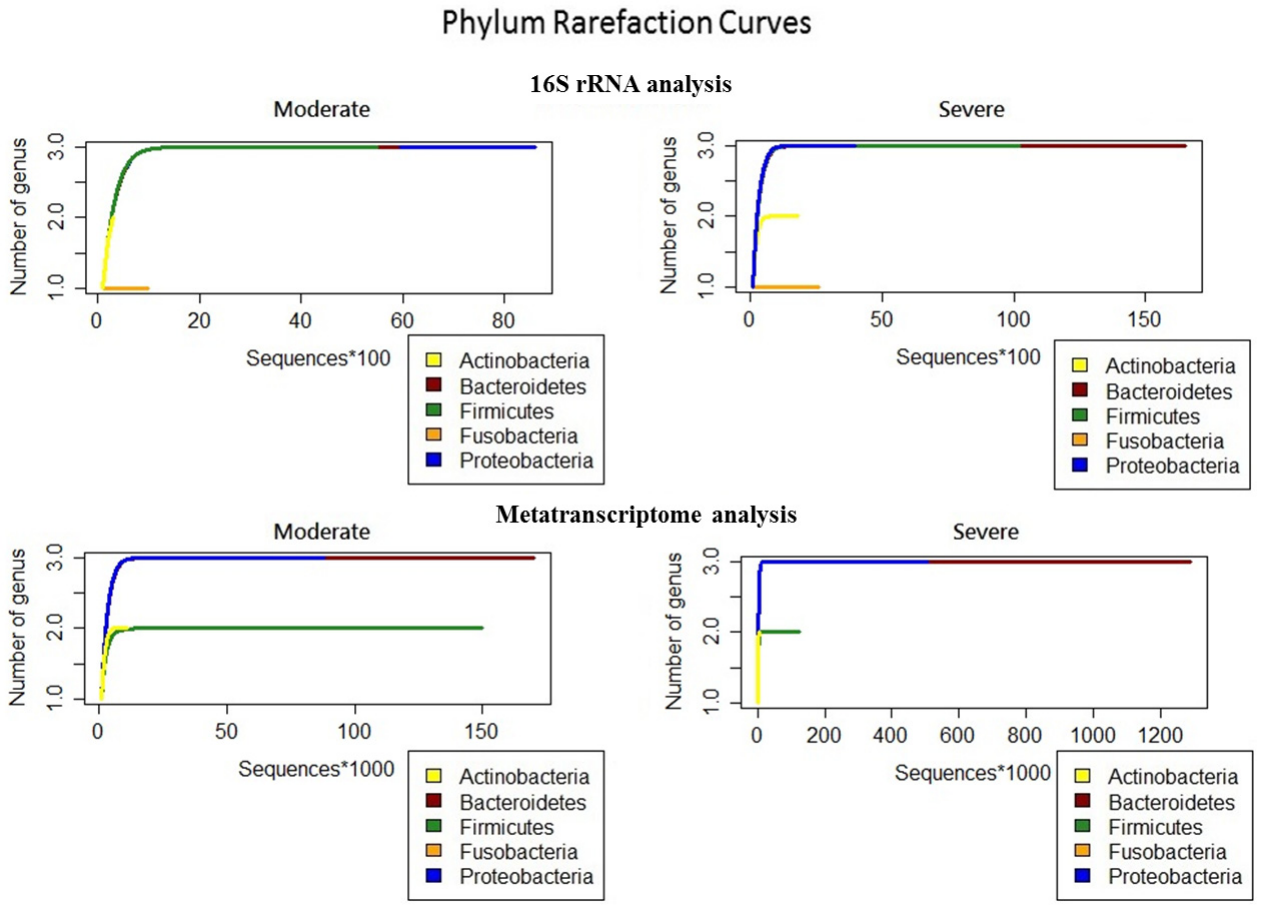
Phylum rarefaction curves for the analyses of 16S rRNA and metatranscriptome unique reads.

The unique reads were found to map to five major phyla: Actinobacteria, Bacteroidetes, Firmicutes, Fusobacteria, and Proteobacteria. To assess our sampling approach and the relative genus richness of each phylum, phylum rarefaction curves were constructed for both the 16S rRNA gene sequence and metatranscriptome datasets (Fig 5). For the 16S rRNA gene sequence data, taxonomic richness at the genus level appeared to be greater in the severe group. The same conclusion could be drawn from the metatranscriptome dataset. Based on the results of unique 16S rRNA gene sequence analysis, Firmicutes, Proteobacteria, and Bacteroidetes were the phyla with the most number of genera. While Proteobacteria appeared to be the dominant phylum in the moderate group, followed by Bacteroidetes, and Firmicutes, Bacteroidetes seemed to be the most abundant in the severe group, with Firmicutes and Proteobacteria being the second and third more dominant phyla. Fusobacteria was the least abundant phylum in both groups. According to the metatranscriptome analysis, the severe samples also showed higher taxonomic richness compared to the moderate group, with more reads mapped to the corresponding genera. In contrast to the 16S rRNA phylum rarefaction result, Bacteroidetes and Proteobacteria were the most enriched phyla in both moderate and severe group, followed by Firmicutes and Actinobacteria. Similar to the 16S rRNA gene sequence dataset, Fusobacteria was the least enriched. Compared to previous analyses based on all high-quality reads, a higher degree of similarity was observed between the 16S rRNA gene sequence and metatranscriptome datasets.

**Fig 6:**
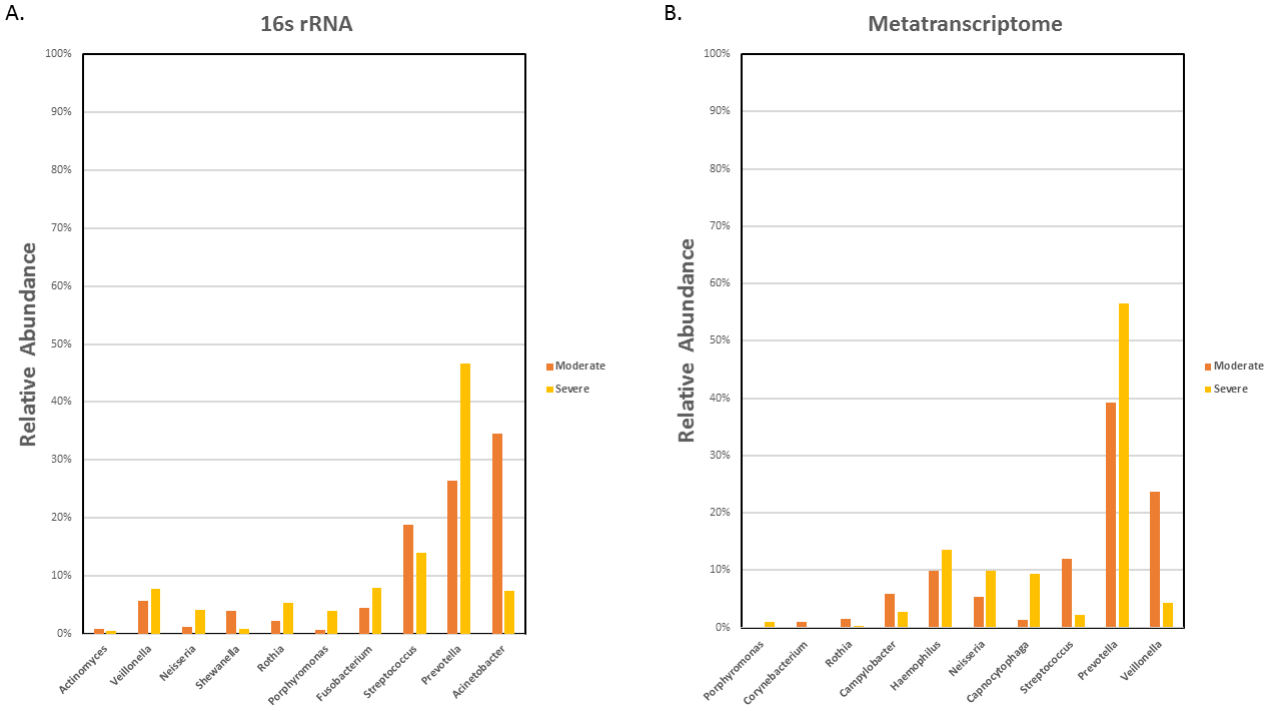
Top 10 genera showing the most difference in relative unique sequence abundance at the genus level in the moderate and severe patients. A) Top 10 most different genera in unique 16S rRNA sequence abundance B) Top 10 most different genera in unique transcript sequence abundance.

With respect to the top 10 genera showing the most differences in percent sequence distribution between the moderate and severe groups, results of the unique sequence analyses also differed from the previous. For this comparison, we further filtered the unique reads based on small coefficient of variation within each group to lessen the effect of individual variations. Based on the 16S rRNA gene sequence dataset, *Acinetobacter*, *Streptococcus*, *Shewanella*, and *Actinomyces* showed higher relative sequence abundance in the moderate group compared to the severe group. In contrast, *Prevotella*, *Fusobacterium*, *Porphyromonas*, *Rothia*, *Neisseria*, and *Veillonella* were more enriched in the severe samples (Fig 6A; S6 Table). Among these genera showing differences in relative bacterial abundance between the two groups, *Shewanella*, *Actinomyces*, and *Veillonella* were absent in the previous top 10 list, whereas *Staphylococcus*, *Bacteroides*, and *Chryseobacterium* that were previously identified to be differentially abundant were not detected when only unique reads were considered. For the unique transcriptome sequences (Fig 6B; S7 Table), discrepancies in the relative abundance among the top ranking 10 genera were greater compared to previous findings, as well as the unique 16S rRNA results. Previously identified *Propionibacterium*, *Pseudomonas*, *Bacillus*, and *Escherichia* did not appear to be differentially abundant, possibly due to the fact that these genera might have been filtered out due to our criteria for minimizing the effect of individual variations. Instead, we observed increased representation of *Campylobacter*, *Corynebacterium*, *Streptococcus*, *Veillonella*, and *Rothia* in the moderate COPD samples. Yet, similar to previous results, *Prevotella*, *Neisseria*, *Haemophilus* and *Porphyromonas* were found to be increased in the severe group. Again, focusing the analysis on unique sequences resulted in increased similarity between the 16S rRNA and metatranscriptome results. Except for disagreement on the relative abundance of *Veillonella* and *Rothia* in the moderate and severe COPD samples, *Porphyromonas*, *Prevotella*, *Fusobacterium*, *Streptococcus*, *Veillonella*, *Rothia* and *Neisseria* were all ranked among the top 10 most different genera by both sequencing analyses. For *Porphyromonas*, *Prevotella*, *Fusobacterium*, *Streptococcus*, and *Neisseria*, candidate species that may contribute to most of the difference in relative abundance in these genera were determined based on the metatranscriptome data (S8 Table). Note that after adjustment for individual variations, differences in relative bacterial abundance between moderate and severe COPD may have become smaller. Thus, although focusing the analysis on group-specific uniquely mapped reads helped individual effects, it seemed to have generated a more conservative picture of the differences in microbiome associated with moderate and severe COPD.

## Discussion

Ranked among the top ten leading causes of death in the world, COPD is becoming increasingly prevalent [3, 4]. Yet, the full spectrum of factors and mechanisms underlying the disease is still not completely understood. As inflammation induced by bacterial infections is thought to play a role in COPD [13], evidence is emerging that variations in the microbiome may be associated with the progression of COPD. However, existing COPD microbiome studies drew conclusions mostly from 16S rRNA gene sequencing data, overlooking the transcriptome profile that may reflect the actual active bacterial communities inhabiting the host tissues.

In this study, we investigated the sputum bacterial microbiome of four moderate and four severe COPD male patients using both 16S rRNA gene sequencing and metatranscriptome sequencing. The identified dominant phyla (Proteobacteria, Firmicutes, Bacterioidetes, Actinobacteria, and Fuscobacteria) represent common bacterial compositions found in the lung [39]. In the moderate COPD sputum samples, we observed increased representations of the Proteobacteria and Firmicutes phyla, and reductions in Bacterioidetes and Fusobacteria compared to the more severe group. Our findings are partially in line with a recent study by Pragman et al. [32], who identified fewer Proteobacteria and more Firmicutes in the bronchoalveolar lavage fluid (BALF) samples of severe COPD patients relative to their moderate counterparts. In an investigation comparing the microbiome profiles of asthma and COPD patients with healthy controls, Hilty et al. [9] also found Bacteroidetes to be reduced in COPD samples, but their study did not specifically looked at COPD severity.

By examining the differential abundance of bacteria at the genus level between the moderate and severe COPD group, we identified similarities and discrepancies between 16S rRNA gene and metatranscriptome sequencing results. With respect to differentially abundant genera between the moderate and severe COPD samples, the two sets of results agreed on five genera—*Prevotella*, *Fusobacterium*, *Porphyromonas*, *Neisseria*, and *Streptococcus*. While 16S rRNA gene sequencing also found *Acinetobacter*, *Chryseobacterium*, *Bacteroides*, *Staphylococcus*, and *Rothia* to differ between the two groups in terms of relative abundance, metatranscriptome sequencing indicated that *Propionibacterium*, *Haemophilus*, *Bacillus*, *Pseudomonas*, and *Escherichia* were also differentially abundant with respect to COPD severity. These discrepancies may be due to the targets of detection. Whereas 16S rRNA gene sequencing provided a general overview of the bacterial communities in the sequenced samples, transcriptome sequencing may have revealed a preliminary picture of the active bacteria in the two COPD severity groups.

Unlike Huang et al. [40] or Hilty et al [9], we did not observe any statistically significant increase in diversity in the more severe group through 16S rRNA16S rRNA gene sequencing. However, we did observed increased diversity associated with severe COPD from analyzing the metatranscriptome data, though this trend was not statistically significant, either. The lack of significant differences between the moderate and severe COPD samples is similar to the study by Erb-Downward et al [41], in which high variations between different lungs tissue samples and individuals were found. Though disagreements between our results and previously reported findings may be due to differences in the sample type, gender, region of sample collection, ethnicity, and more importantly, we suspected that individual variations may also affect our investigation, especially when the sample size was small.

To minimize influences from individual variations, we focused subsequent analyses on reads mapped to one specific species and genus in the metatranscriptome data and 16S rRNA gene sequence data, respectively. These reads were also common to all samples within a specific group and showed small variations among samples of the same COPD severity. Mapped reads that occurred in only one sample of a group were discarded in this analysis. Moderate and severe COPD samples shared more similarities in terms of relative bacterial abundance at the phylum level, but differences were still observed at the genus level. The 16S rRNA and metatranscriptome datasets agreed on *Porphyromonas*, *Prevotella*, *Streptococcus*, *Veillonella*, *Rothia* and *Neisseria* being among the differentially abundant genera between the moderate and severe COPD samples. On the other hand, whereas 16S rRNA gene sequence analysis showed *Acinetobacter*, *Shewanella*, *Actinomyces*, *Fusobacterium* to differ in abundance relative to COPD severity, metatranscriptome sequencing identified *Campylobacter*, *Corynebacterium*, *Capnocytophaga*, and *Haemophilus* to be different in representation in the two COPD groups. We believe by reducing individual effects, our analyses may offer a more reliable picture of the microbial differences associated with COPD severity in the context of our small sample size. Therefore, for subsequent discussions, we decided to focus on the results of unique sequence analyses.

Though the two sequencing methods still disagreed with regard to the relative representation of these genera in the two groups, this analysis identified differences in several pulmonary inhabitants and pathogens, *Prevotella* and *Rothia*, *Streptococcus*, and *Porphyromonas*, that have also been observed in other studies [32, 41]. It is likely that the number of these bacteria may be particularly important to the disease progression of COPD. In fact, these common pulmonary inhabitants and pathogens found to be differentially represented in the severe and moderate COPD groups have been associated with lung inflammation or diseases. For instance, a 16S rRNA gene sequencing study suggested that high abundance of *Prevotella* and *Veillonella* may enhance pulmonary inflammation [42]. Our study revealed that these genera may indeed play a role in COPD. Moreover, transcriptome sequencing found *Veillonella* to be more highly represented in the moderate COPD group, and *Prevotella*, increased in the severe COPD patients’ sputum, suggesting that alterations in the number of actively transcribing *Prevotella* and *Veillonella* may underlie the differences between moderate and severe COPD.

The potential involvement of common oral bacteria *Fusobacterium* and *Porphyromonas* in chronic respiratory diseases has been implicated in many recent studies. An association between poor periodontal health and COPD was noted in two large health surveys [43, 44], and periodontitis was found to be able to increase the risk of COPD by 60%, exacerbating the disease condition [45, 46]. The hypothesis that oral hygiene may influence the susceptibility or severity of COPD is supported by both our study and existing evidence [32, 41, 47], in which *Fusobacterium* and *Porphyromonas*, central to the pathogenesis of periodontitis [48], were found to be differentially abundant among those with differing COPD severity or between healthy controls and COPD patients. It is difficult to conclude at this point whether these oral pathogens are directly related to COPD or just a general reflection of poor health behaviors among COPD patients.

Based on our observation of *Fusobacterium* and *Porphyromonas* being more highly represented in the moderate group, we suspect that these bacteria may increase the risk of oral inflammation, thereby weakening the overall immune defense in the naso- or oropharyngeal airways and indirectly contributing to chronic inflammations in COPD. Yet, in severe COPD, other dominant bacterial species, perhaps belonging to *Streptococcus*, *Haemophilus*, or *Neisseria*, may take over to modulate the disease progression. In fact, species under the genera *Streptococcus* and *Haemophilus* are often present in cases of severe COPD and exacerbations [49, 50]. Increased number of *Haemophilus* and *Streptococcus* bacteria have also been detected in COPD patients relative to healthy control subjects [51]. This is in line with our observation of the greater abundance of *Streptococcus* and *Haemophilus* bacteria in moderate and severe COPD, respectively. In particular, we found *Haemophilus parainfluenza*, a common commensal organism in the oropharynx, as well as *Haemophilus influenza*, both of which have been suggested to play pathogenic roles in COPD [52], to be highly abundant in severe COPD compared to their moderate counterparts. Metatranscriptome sequencing indicated that among the *Streptococcus* species that were more highly represented in moderate COPD sputum, common oral inhabitants like *Streptococcus sanguinis*, *Streptococcus salivarius*, and *Streptococcus parasanguinis* showed the greatest differences between moderate and severe COPD, while *Streptococcus pneumoniae*, the well-known culprit of exacerbations [50], showed a slight difference between the two severity states. Poor periodontal health is increasingly being recognized as a contributor to respiratory inflammation and exacerbations in COPD patients, as oral bacteria may be carried into the lung and cause infections [53, 54]. Our findings in the COPD sputum provide further support for the association between the oral microbiota and disease progression of COPD. While the pathogenic role of *Neisseria* in COPD is unclear, *Neisseria meningitides* is known to cause respiratory infections [55], periodontal diseases [56] and lung abscess [57], respectively. We speculate that their increased abundance may also induce inflammatory responses in patients with severe COPD.

In this study, we have presented the bacterial profiles of male patients with moderate and severe COPD based on 16S rRNA gene and transcriptome sequencing. We showed that the two methods differ in their ways of enriching our understanding of COPD. Whereas the former provided an overview of the potential microbial biomarkers associated with COPD severity, the latter offered insights into the active species that may potentially control the disease progression of COPD. Note that some of the differentially abundant species identified from 16S rRNA gene sequencing have been detected as potential reagent contaminants [58]. In the library preparation process for 16S rRNA gene sequencing, we used a negative control in an attempt to reduce sequencing noises contributed by reagents. The dominant species in the negative control were found to be different from those in the clinical samples, in addition to being extremely rare in the latter groups. Yet, we still observed two known reagent contaminants, *Streptococcus* and *Acinetobacter*, as being differentially represented in moderate and severe COPD. This might indicate that although species of these two genera were indeed present in the reagents, their existence in sputum might also be relevant to COPD severity. Finally, our study is limited by the lack of negative controls for the extraction process. Thus, influences of potential contaminants from the extraction reagents may have been overlooked.

To our knowledge, our study should be the first, or at least among a few, that has presented a metatranscriptome map of the bacterial communities in COPD. However, several limitations should be noted. First, the small sample size in our study may have prohibited us from observing more significant differences between the moderate and severe group. Although by focusing our analysis on unique reads shared by all individuals of the same group, we were able to observe common trends that are in line with existing studies, important microbial signatures associated with COPD may have been overlooked. Since region of sample collection, gender, disease condition, medication, and tissue types may affect the findings, our results should be interpreted in the context of sputum samples collected from Taiwanese males receiving treatment for moderate and severe COPD. Therefore, we may have described the microbiome of moderate and severe COPD, and demonstrated the importance of investigating the transcriptomes of the microbial species involved in the disease, but much work remains to enhance our understanding of the lung microbiome in COPD.

## Supporting Information

S1 Table Clinical data of COPD patients. (XLSX)

S2 Table 16S rRNA sequence distribution among different taxonomic classes. (XLSX)

S3 Table 16S rRNA sequence distribution differences between the moderate and severe COPD group at the genus level. (XLSX)

S4 Table Distribution of sequenced RNA reads among different taxonomic classes. (XLSX)

S5 Table Metatranscriptome sequence distribution differences between the moderate and severe COPD group at the genus level. (XLSX)

S6 Table Unique 16S rRNA sequence distribution differences between the moderate and severe COPD group at the genus level. (XLSX)

S7 Table Unique metatranscriptome sequence distribution differences between the moderate and severe COPD group at the genus level. (XLSX)

S8 Table Differentially abundant species in *Porphyromonas*, *Streptococcus*, *Fusobacterium*, *Neisseria*, and *Haemophilus* between moderate and severe COPD. (XLSX)
